# What is “eczema”?

**DOI:** 10.1111/1346-8138.17439

**Published:** 2024-09-20

**Authors:** Yoshiki Tokura, Marina Yunoki, Shumpei Kondo, Masaki Otsuka

**Affiliations:** ^1^ Department of Dermatology and Skin Oncology Chutoen General Medical Center Kakegawa Japan; ^2^ Allergic Disease Research Center Chutoen General Medical Center Kakegawa Japan

**Keywords:** eczema triangle, history, hyperpigmentation, innate lymphoid cell, T cell

## Abstract

Eczema is the most common category of inflammatory skin disorders as dermatologists see many patients with eczematous diseases in daily practice. It is characterized by the three major morphological features: multiple‐pinpoint condition, polymorphism, and itch. To describe polymorphism, “eczema triangle” has been used in German/Japanese dermatology. The multiple pinpoints correspond to numerous tiny foci from which individual papules/vesicles arise. The polymorphism betrays composition of erythema, papule, seropapule, vesicle, pustule, scale, and crust, which are seen in acute eczema. Meanwhile, chronic eczema is represented by lichenification and hyperpigmentation, and possibly by hypopigmentation. In acute eczema, spongiosis is associated with overproduction of hyaluronic acid, secretion of self‐protective galectin‐7, and decreased expression of E‐cadherin. In the upper dermis, Th1/Tc1 or Th2/Tc2, and additional Th17, Th22, and/or Tc22 infiltrate, depending on each eczematous disease. Innate lymphoid cells are also involved in the formation of eczema. In chronic eczema, periostin contributes to remodeling of inflammatory skin with dermal fibrosis, and epidermal melanogenesis and dermal pigment deposition result in hyperpigmentation. Finally, eczematous diseases are potentially associated with increased risk of comorbidities, including not only other allergic diseases but also coronary heart disease and mental problems such as depression. Although the original word for eczema is derived from old Greek “ekzein,” eczema remains a major target of modern science and novel therapies.

## 1 INTRODUCTION

Eczema is the most common category of inflammatory skin disorders as dermatologists see many patients with the diseases classified into this group in daily medical practice. While eczema is frequently used to communicate among dermatologists, they may not exactly know the origin and history of the term “eczema.” The original word for eczema is “ekzein (Greek),” which means “boil over” or “break out” (Oxford Learner's Dictionaries). Before eczema earned its formal title, descriptions of similar skin conditions appeared in ancient Egyptian texts. The Ebers Papyrus, one of the earliest known medical documents, was written more than 3000 years ago, and skin issues were described in this ancient writing.^1^ Hippocrates also contributed to theories on the origins and treatment of eczema‐like skin conditions around 400 bc.^2^ Eventually, eczema was coined in 1817 to describe a fluid‐filled, blistering rash–like sunburn.^3^ Thus, the original meaning represents aggressive acute eczema and does not match the currently used eczema that we typically image today.^4^

Despite its highly frequent usage, “eczema” has been terminologically confused with “dermatitis.” Eczema and dermatitis are often used synonymously to denote a polymorphic pattern of inflammation of the skin characterized, at least in its acute phase, by erythema, vesiculation, and pruritus.^5^ One of the representative diseases lacking standardized nomenclature using eczema/dermatitis is atopic dermatitis (AD) or atopic eczema.^6^ The term atopic “dermatitis” was rarely used until the late 1970s, after which AD became commonly used as compared with atopic “eczema” and continuously increased until 2015. Atopic eczema decreased between 2008 and 2015. One may recognize that “eczema” is used for skin features as seen in childhood eczema, flexural eczema, infantile eczema, dyshidrotic eczema, and nummular eczema.^6^ Meanwhile, “dermatitis” is intended to be used for disease names, such as contact dermatitis and seborrheic dermatitis.

Although it takes courage to describe the whole aspects of eczema, in this review article, we aim to reveal the classical concept and current etiological thoughts of eczema. We review the morphology of eczema, looking back on the “eczema triangle,” and discuss acute and chronic eczema, focusing on the pathophysiology of spongiosis and fibrosis, respectively.

## 2 MORPHOLOGY OF ECZEMA

### 2.1 Three features of eczema

Among inflammatory skin diseases, eczema has characteristic clinical features. However, the phenotype of eczema is not homogeneous, and it exhibits various appearances. The skin comprises the three layers, including the epidermis, dermis, and subcutaneous fat tissue. In inflammatory skin diseases, one or more of these three layers are affected. When the epidermis is inflamed, papules, vesicles, pustules, erosion, scale, and other primary or secondary elements appear and are called “epidermal changes.” When the dermis is affected, erythema arises because of telangiectasia of inflamed vessels. When the subcutis undergoes inflammation, induration is clinically palpable. In inflammatory skin diseases, the combination of the epidermal and dermal changes is the most frequently seen pattern, as observed in the three representative diseases including eczema, psoriasis, and lichen planus.^7^

Eczema is characterized by the three major morphological features: multiple‐pinpoint condition, polymorphism, and itch.^5^ The multiple‐pinpoint condition is derived from numerous tiny foci where individual papules/vesicles arise. The polymorphism betrays composition of various elements, such as erythema, papule, seropapule, vesicle, pustule, scale, and crust. Itch is remarkable in eczema and more severe than those of psoriasis and lichen planus.

**FIGURE 1:**
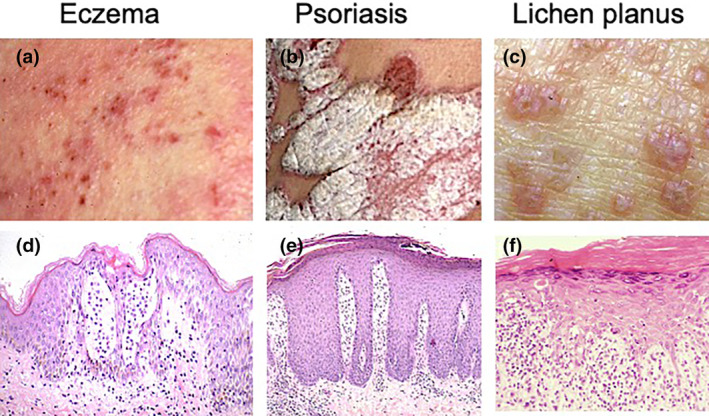
Three major diseases affecting the epidermis and dermis. Clinical pictures and histopathologies of eczema (a, d), psoriasis vulgaris (b, e), and lichen planus (c, f).

Eczema is especially unique in its polymorphism (Figure 1a) compared with psoriasis and lichen planus. Psoriatic plaque is well‐demarcated erythema with a thick scale (Figure 1b), and lichen planus exhibits violaceous red papules (Figure 1c). Thus, psoriasis and lichen planus display rather monotonous appearances. The polymorphic appearance of eczema makes its diagnosis difficult, compared with the other two diseases.

### 2.2 Eczema Triangle

In most Japanese textbooks on dermatology, the eczema triangle is presented in the chapter on eczema/dermatitis. On the contrary, this pattern is not typically depicted in American dermatology textbooks. In a famous German textbook by Braun‐Falco, Plewig, and Wolff,^8^ a similar eczema triangle is described, which looks like a precedent to the Japanese triangle. Given the historical importance of German medicine to Japanese medicine, the eczema triangle is considered to originate from German or related dermatology. The triangle can date back to 1904, when it was described in a dermatology textbook written by Kreibich in Vienna,^9^ who later became a professor at Prague University. Thus, it is assumed that the eczema triangle came to Japan from Vienna/Prague through Germany. Since the eczema triangle appeared in a Japanese textbook in 1940,^10^ we can trace its history back to such early days of westernized Japanese medicine.

**FIGURE 2:**
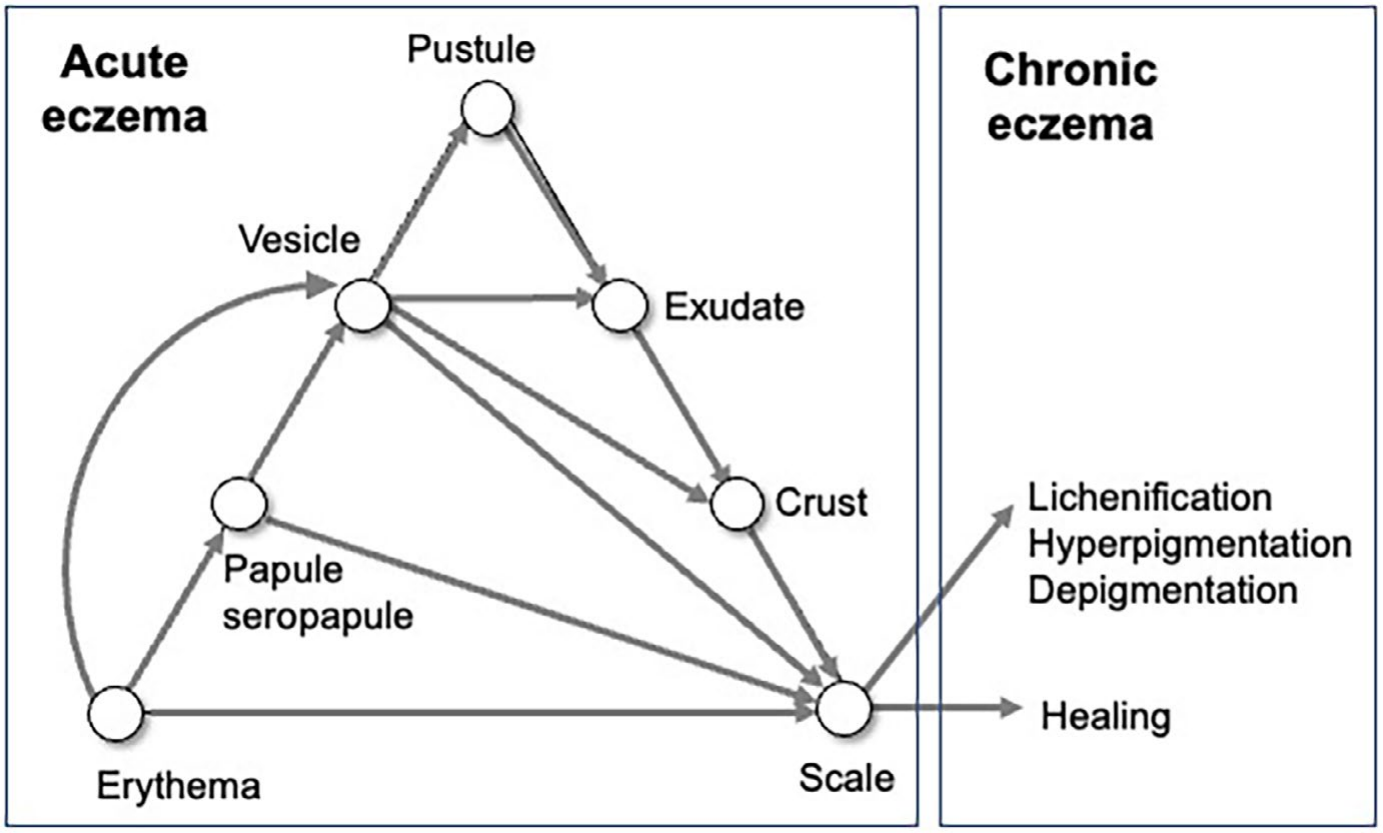
The sequential evolution of eczema with epidermal changes in the eczema triangle.

In the eczema triangle, one can observe the sequential evolution of eczema with epidermal changes (Figure 2).^11^ Erythema progresses to papules and seropapules, which further evolve into vesicles and pustules. They are followed by exudative alteration and resultant crust formation. The eruption finally heals or progresses to lichenification.

It should be noted that these changes occur in individual pinpoint lesions. When they begin at the same time, multiple pinpoints may follow the same chronologic evolution process. Acute contact dermatitis may show such synchronized development, since a high number of pinpoints emerge in a certain area upon exposure to a contactant. In most cases of eczema, however, the initial timepoint of each pinpoint is different, resulting in a chaotic appearance composed of papules, vesicles, scale, and crust. Thus, individual pinpoints arise in a nonsynchronized fashion, giving rise to polymorphism in an eczematous area.

## 3 ACUTE AND CHRONIC ECZEMA

### 3.1 Definition

There has been no clear definition of acute and chronic eczema. Since this terminology reflects chronological changes, chronic eczema should be considered a progression from acute eczema over time. However, dermatologists can diagnose a given lesion as “acute” or “chronic” by inspection on the first examination without knowing the exact time course. Thus, acute and chronic eczema is usually determined based on morphology rather than chronology.

The eczema triangle basically represents acute eczema. In some of the models of the eczema triangle that were historically modified, chronic lesions, such as lichenification, have been modestly included in parallel with healing. In the eczema triangle from the textbook written by Braun‐Falco, Plewig, and Wolff,^8^ only “resterythem” (rest erythema) was given as sequela. Probably, the chronic lesions may have been out of the scope of the eczema triangle in its original form. However, lichenification, hyperpigmentation, and/or depigmentation are necessary as possible lesions to terminate eczema (Figure 2).

### 3.2 Polymorphism in acute and chronic eczema

**FIGURE 3:**
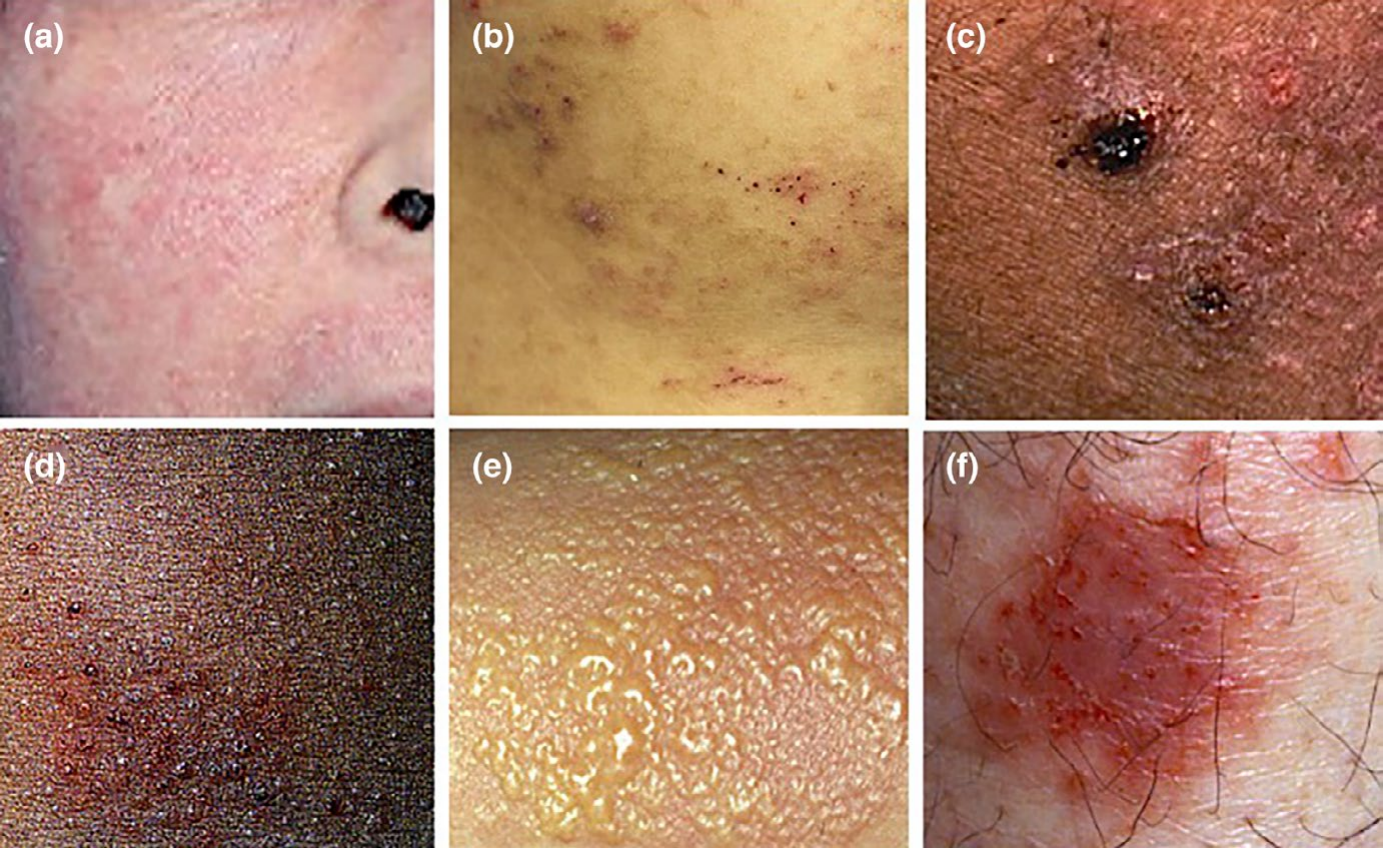
Polymorphism in acute eczema. (a) Multiple pinpoint papules with scale on the erythematous background. (b) Various‐sized papules with crust and scale. (c) Multiple papules with marked erosion and crust on the erythematous background. (d) Relatively monotonous papules with erythema and scale. (e) Vesicles predominating with erythema. (f) Erosive small papules on the erythematous area. Original magnification, x100 (d‐f).

When we see the clinical appearance of acute eczema, we recognize that the polymorphism of eczema has two meanings. The first one is the original idea and was mentioned above. The pinpoints exhibiting different elements, including papule, vesicle, erosion, scale, and crust, are intermingled with each other in the same eczematous area (Figure 3a–c). Occasionally, the pinpoints are less polymorphic with the predominance of papules (Figure 3d), vesicles (Figure 3e), or erosive papules (Figure 3f). This may happen when individual pinpoints start at the same time, and they are synchronized thereafter.

**FIGURE 4:**
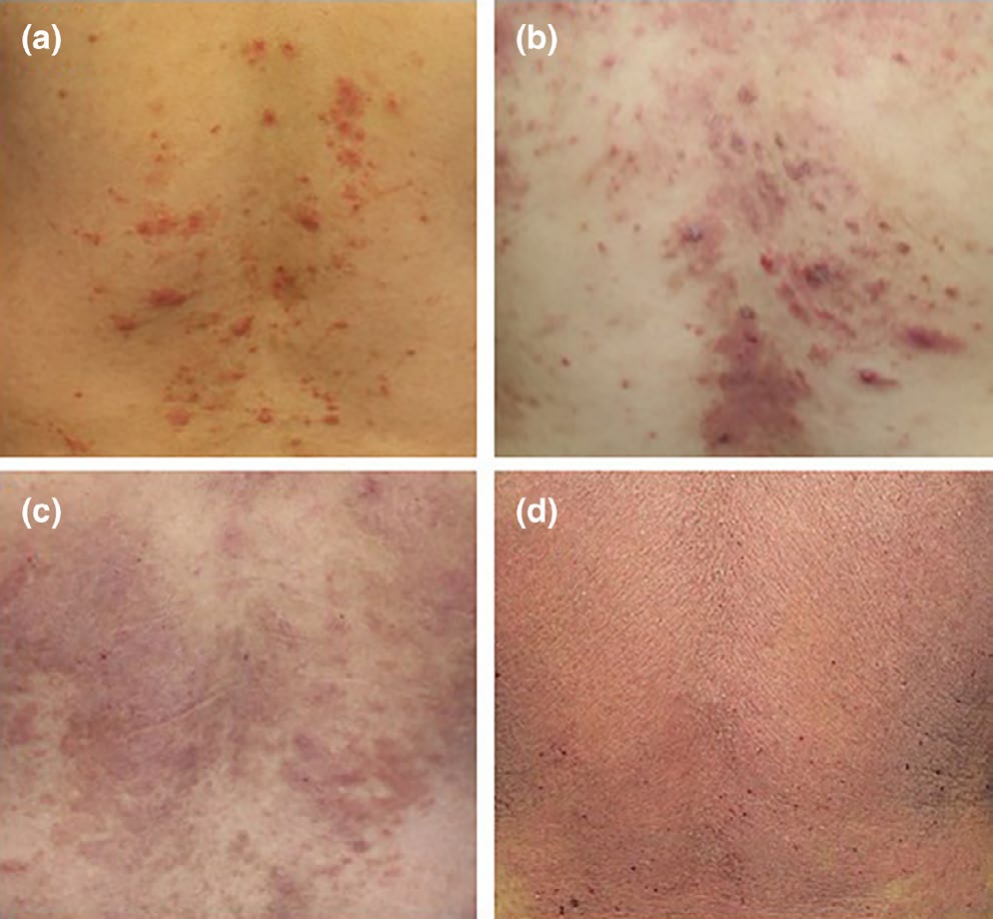
Polymorphism with individual differences on the back of patients with atopic dermatitis. (a, b) Small to medium papules/nodules. (c) Coalesced papules/nodules evolving into plaques. (d) Papular eruption further diffusely extending to form erythrodermic appearance.

The other meaning of polymorphism is individual patient variability. For example, AD shows polymorphism in the eruption occurring even on the same site among individual patients. As shown in the back lesions of AD, papules/nodules are various in size from case to case (Figure 4a,b). Some papules/nodules are coalesced, evolving into plaques (Figure 4c). Papular eruptions further diffusely extend to form an erythrodermic appearance (Figure 4d). Thus, polymorphism based on the individual variability is seen in identical eczematous diseases. There are many factors to induce individual differences associated with polymorphism. Although barrier condition and allergen type are the common causative factors for AD, different eczematous eruptions can occur even in patients with *FLG*‐mutated AD who are exposed to the same allergens. In addition, the anatomical sites may phenotypically affect eczematous lesions, as represented by palmoplantar eruptions with pompholyx, hyperkeratosis, and fissuring.

**FIGURE 5:**
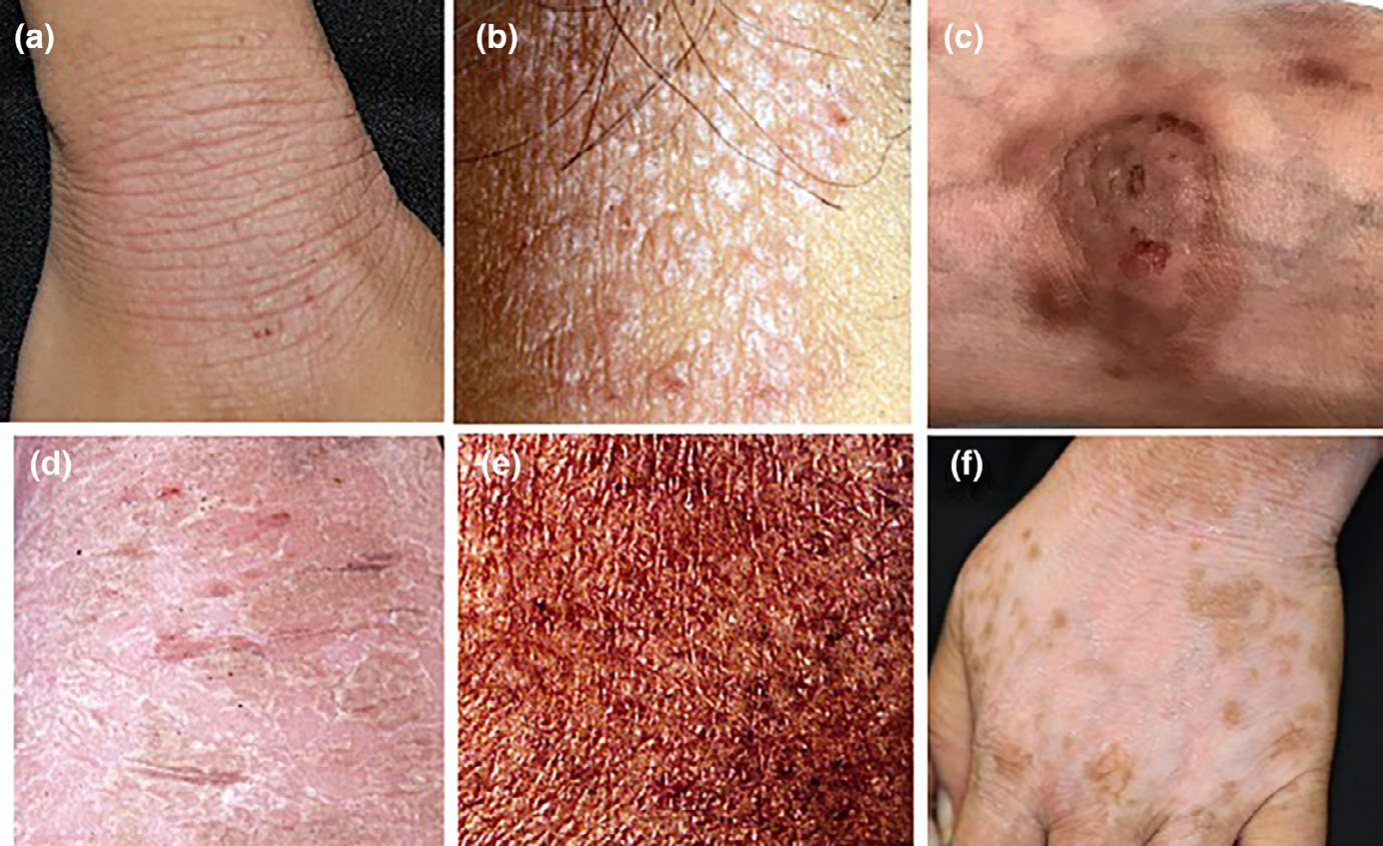
Polymorphism in chronic eczema. (a) Lichenification on the dorsum of the wrist. (b) Lichenification on the nuchal region, called lichen simplex Vidal. (c) Large lichenified nodule on the dorsum of the foot. (d) Fissures within lichenified lesion on the sole. (e) Pigmentation known as the dirty neck in atopic dermatitis. (f) Depigmentation on the dorsum of the hand.

In chronic eczema, the fresh papular/vesicular elements disappear. Instead, hard texture and scaling, called lichenification (Figure 5a,b), take place as a result of histopathological changes, i.e. hyperkeratosis, epidermal acanthosis, and dermal fibrosis. The nuchal lichenification is called lichen simplex Vidal (Figure 5b). When the patients scratch certain lesions extensively, they develop large lichenified nodules (Figure 5c). Fissures within lichenified, hyperkeratotic areas are observed especially on the palms and soles (Figure 5d). Hyperpigmentation is another well‐known chronic manifestation as it presents as the “dirty neck” in AD (Figure 5e). Depigmentation is occasionally observed along with chronic lesions (Figure 5f). It is considered that depigmented lesions in AD belong to leukoderma or vitiligo, and 15% of patients with AD exhibit such depigmentation.^12^

## 4 CLASSIFICATION OF ECZEMA

Eczema has been classified into many diseases from different points of view (Table 1). Categorization of eczema is not completely harmonious because the classification viewpoints overlap in some of the diseases. In most diseases, the cause and pathogenesis are major points for classification. In eczema, such diseases include contact dermatitis, photocontact dermatitis, asteatotic eczema, stasis dermatitis, and genetic dermatoses. However, there are many conditions in which causative agents are clinically obscure.

**TABLE 1:** Classification of eczema.

1. Clear causes and etiologies
(1) Contact dermatitis
a. Irritant contact dermatitis
b. Allergic contact dermatitis
(2) Photocontact dermatitis
a. Phototoxic contact dermatitis
b. Photoallergic contact dermatitis
(3) Atopic dermatitis
(4) Asteatotic eczema
(5) Stasis dermatitis
2. Clinical course
(1) Acute eczema
(2) Chronic eczema
3. Body sites
(1) Hand eczema
(2) Diaper dermatitis
4. Specified eczema
(1) Nummular eczema
(2) Autosensitization dermatitis
(3) Systemic contact dermatitis
(4) Seborrheic dermatitis
5. Genodermatosis
(1) Netherton syndrome
(2) Peeling skin syndrome type B
(3) SAM syndrome
(4) NISCH syndrome
(5) Wiskott‐Aldrich syndrome
(6) Hyper‐IgE syndrome

In the eczema spectrum, it is difficult to classify AD into an appropriate subcategory, because it encompasses different viewpoints. In the Table 1, we categorize AD into “clear causes and etiologies,” but other categories may also accommodate AD. Since AD includes exudative eczema and lichen simplex Vidal, it belongs to both acute and chronic “clinical course” categories. Since the majority of patients with AD experience palmar lesions, hand eczema in “body sites” implies AD. Patients with AD show nummular eczema in “specified eczema” following insect bites.

Historically, AD has been divided into several classical subtypes.^13^ Accordingly, recent studies have demonstrated that AD has different endotypes.^14^, ^15^ One of the standpoints for AD endotyping is the stratum corneum barrier condition, represented by filaggrin (FLG) deficiency and caused by the loss‐of‐function mutation of the *FLG* gene.^16^ However, we cannot classify AD as “genodermatosis,” because not all (one‐fourth) Japanese patients with AD have *FLG* mutations and other factors are also related to AD pathology. Recent findings on endotyping of AD have contributed to understanding the heterogeneity of AD.^14^, ^15^ In contrast, Netherton syndrome is a genodermatosis, because only the *SPINK5* mutation and resultant serine protease activation induces AD‐like eczematous lesions.^17^ Finally, some patients with asteatotic eczema share *FLG* mutations with AD, suggesting that those patients may be diagnosed with senile AD.

Thus, AD encompasses several categories other than AD, implying the difficulty in categorization of some eczematous diseases.

## 5 OTHER SKIN DISEASES RELATED TO ECZEMA

### 5.1 Dyshidrotic eczema and palmoplantar pustulosis

The palms and soles are unique sites where eczema manifests pompholyx or vesicles. Pompholyx is characterized by recurrent crops of vesicles on the lateral aspects of the fingers and the palms and soles.^18^ Dyshidrotic eczema shares the predilection sites with palmoplantar pustulosis (PPP). Moreover, vesicles precede pustules in the early stage of PPP. Thus, there are clinical similarities between dyshidrotic eczema and PPP. To differentiate dyshidrotic eczema from PPP, several observations have been reported.^19^, ^20^, ^21^ Histopathologically, spongiotic vesicles indicate dyshidrotic eczema, while neutrophilic microabscesses suggest PPP. In PPP, however, it should be noted that neutrophilic microabscesses are seen only in pustulo‐vesicles or immature pustules.^19^, ^20^, ^21^ Additionally, in dyshidrotic eczema, epidermal keratinocytes contain a high amount of hyaluronate,^21^ which is associated with the development of spongiosis.^22^

Although patients with PPP usually do not show circulating neutrophilia, they have higher levels of neutrophil extracellular traps in the peripheral blood, indicative of neutrophil activation.^23^ Consistently, interleukin (IL) 8 and IL‐22 were also elevated. Therefore, PPP shows activated neutrophils and similar cytokine preponderance to psoriasis. While PPP is estimated to be palmoplantar pustular bacterid, it also may be considered as palmoplantar pustular psoriasis.^24^, ^25^ Accordingly, patients with PPP occasionally have plaque psoriasis. In this respect, dyshidrotic eczema is different from PPP in the pathogenesis, although clinical similarities are seen between the two conditions.

### 5.2 Prurigo chronica multiformis

This nomenclature is often used in Japan. The Japanese clinical guidelines for chronic prurigo were published in 2012 to reduce confusion regarding the concepts of prurigo and to standardize laboratory tests and treatments. The diagnostic terms and each definition have been changed over time in prurigo, and new therapies for prurigo nodularis have currently been approved. We thus updated and revised the guidelines to classify prurigo based on the clinical appearances and causes. The subtypes of prurigo were simplified, including prurigo nodularis, prurigo chronica multiformis, and prurigo, not otherwise specified.^26^ There was a consensus on prurigo chronica multiformis by Japanese committee members of prurigo guidelines, although no description was noted on this entity in American textbooks.

In prurigo chronica multiformis, the papules are relatively large but smaller than those of prurigo nodularis. The papules typically occur on the abdomen but can be seen on other sites of the trunk and limbs. The eruption is composed of relatively large papules, but eczematous elements may be intermingled with prurigo. Since one can occasionally appreciate positive dermographism, urticaria is thought to be present as the background of the condition.^27^ This suggests that scratching following wheal induces both prurigo and eczema, forming prurigo chronica multiformis.

### 5.3 Papuloerythroderma

Papuloerythroderma, described by Ofuji et al.,^28^ initiates with multiple solid papules, which are coalesced gradually to form erythroderma. It was originally reported to be associated with internal malignancies. Recent studies, however, have shown that it is a manifestation of cutaneous T‐cell lymphoma^29^ or a type of drug eruption.^30^, ^31^, ^32^

In cases of drug eruption, it is revealed that papuloderma is evoked by the causative drug, but the eruption cannot be completely alleviated by its discontinuation. Moreover, in some patients, an eczematous eruption is antecedent to drug eruption, and drug intake exacerbates the eruption, evolving into papuloerythroderma.^31^, ^32^ It was found that the number of circulating Th2 cells is markedly increased in patients with papuloerythroderma.^32^ There is a notable tendency for the lymphocyte transformation to test positive to the culprit drug but negative to the patch test, suggesting that reactive T cells are Th2 cells rather than Th1 cells.^32^ This suggests that drug‐induced papuloerythroderma is mediated by Th2 cells infiltrating in the precedent eczema. Accordingly, such a Th2 skewing condition is also seen in papuloerythroderma of cutaneous T‐cell lymphoma.

### 5.4 Eczematide (eczematid)

This term appears to be derived from French dermatology and not well characterized in other countries. However, it may be used in daily clinics even in Japan to denote a certain eczematous condition. Eczematides or eczematids are itching dermatoses showing indistinct redness and pityriasiform scales^33^ and share features with pityriasis rosea Gibert, pityriasis lichenoides (subacute or chronica), patch parapsoriasis, and seborrheic dermatitis. Some infections may trigger this condition and autosensitization may even occur. Meanwhile, eczematid‐like purpura of Doucas‐Kapetanakis is proposed as a subtype of chronic pigmentary purpura.^34^

## 6 PATHOGENESIS OF ECZEMA

### 6.1 Histopathology

Acute eczema is characterized by epidermal and dermal changes. In the epidermis, there is intercellular edema, which has been coined spongiosis. Aggravation of spongiosis results in retention of a high amount of fluid in the intercellular space, forming spongiotic vesicles (Figure 1d). In the upper dermis, perivascular infiltration of T cells is noted. These features are different from those of psoriasis, characterized by elongation of rete ridges, parakeratosis, and Munro microabscesses (Figure 1e), and from those of lichen planus, characterized by hyperkeratosis with hypergranulosis, band‐like infiltration of T cells and basal cell degeneration with Civatte bodies (Figure 1f).

In chronic eczema, keratinocytes proliferate, exhibiting epidermal acanthosis, and fibroblasts are activated, showing remodeling with fibrosis. Lymphocyte infiltration persists, although to a lesser degree than in acute eczema. Additionally, there may be postinflammatory hyperpigmentation, which comprises epidermal melanosis and dermal melanophage formation.

### 6.2 T‐cell populations and innate lymphoid cell populations

Th1 and Th2 cells were defined in 1986–1987 based on the different cytokine profiles they produce.^35^, ^36^ Th1 cells produce interferon (IFN) γ and IL‐2, and Th2 cells secrete IL‐4, IL‐5, IL‐10, IL‐13, and IL‐31.^37^ Subsequently, it was found that Th17 cells, producing IL‐17A/F and IL‐22, are an important subset in defense systems^38^ and certain diseases, in particular, psoriasis.^39^ In addition to these CD4^+^ T cells, CD8^+^ T cells with the similar cytokine profiles are denoted to be Tc1, Tc2, and Tc17, respectively.^40^

More recently, innate lymphoid cells (ILCs), possessing no T‐cell receptors, play critical roles in the peripheral tissues.^41^ Three ILC populations are categorized as ILC1, ILC2, and ILC3. Their cytokine profiles correspond to Th1, Th2, and Th17, respectively.^41^ The terms type 1 and type 2 immunity or inflammation, induced by Th1/Tc1/ILC1 and Th2/Tc2/ILC2, respectively, are widely accepted. In addition, the immunity evoked by Th17/Tc17/ILC3 is named type 3 immunity or inflammation in this article, which is also called type 17 immunity. Since IL‐17 and/or IL‐22 can be secreted not only by Th17 but also by Th22 and Tc22,^42^ they are involved in type 3 inflammation.

**FIGURE 6:**
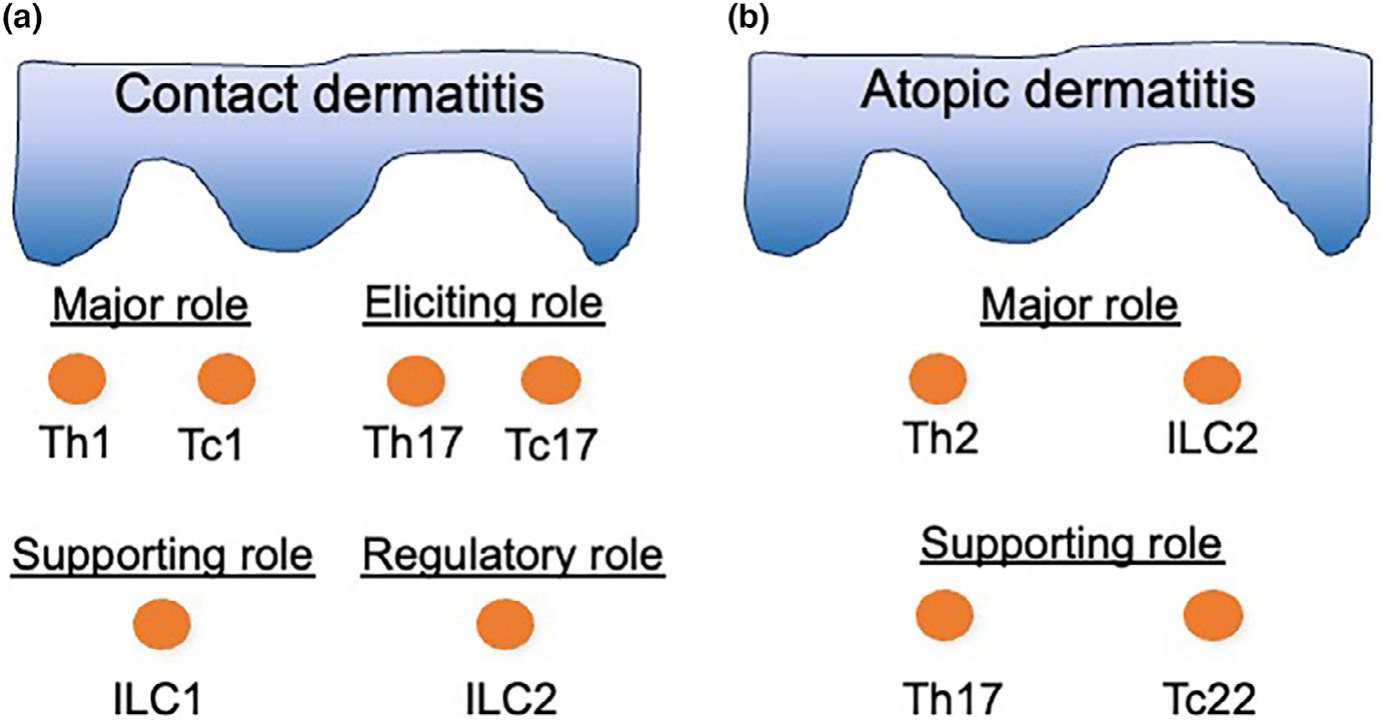
T‐cell and ILC populations in eczema. T cells and innate lymphoid cells (ILCs) infiltrating in contact dermatitis (a) and atopic dermatitis (b) and their roles are depicted based on references 43, 44, 45, 46, 47, 48, 49, 50, 51, 52, 53, 54, 55, 56, 57, 58, 59, 60, 61, 62, 63, 64, 65

Depending on each eczematous disease, different T‐cell subsets infiltrate in the dermis. In contact dermatitis (Figure 6a), both CD4^+^ and CD8^+^ T cells, corresponding to Th1 and Tc1, respectively, infiltrate in the upper dermis.^43^ Furthermore, it was found that Th17 and Tc17 are present and play a role in the elicitation of contact hypersensitivity.^44^ More recently, participation of ILCs in allergic contact dermatitis has been found and ILC populations interact with each other.^45^, ^46^ More specifically, lack of ILC2 in type 1–dominated contact hypersensitivity results in enhanced inflammation, suggesting a regulatory role of ILC2 in this context.^46^

AD is a representative type 2 inflammation^47^ induced by Th2^48^, ^49^ and ILC2^50^, ^51^ (Figure 6b). Their cytokines IL‐4 and IL‐13 play a central role in the pathogenesis.^52^, ^53^, ^54^ The target cells include Th2 per se, basophils, mast cells, eosinophils, M2 macrophages, keratinocytes, fibroblasts, and smooth muscle cells.^56^, ^57^, ^58^ Thus, there are autocrine systems influencing the surrounding cells. It is notable that IL‐4 is produced by basophils by virtue of IL‐33 in the initial step.^45^ AD pathogenesis also remarkably involves ILC2.^59^, ^60^, ^61^, ^62^, ^63^ Accumulated evidence indicates that ILC2 markedly produces IL‐13, elaborates IL‐4 in a certain condition, and serves as a more efficient IL‐13 producer than do Th2 cells.^59^, ^60^, ^61^, ^62^, ^63^ In addition, Th17 and Tc22 cells participate in the occurrence of AD^64^, ^65^.

Since both contact dermatitis and AD present as eczema, it follows that eczema can be induced by any of type 1 and type 2 immunity; moreover, type 3 immunity can support the inflammatory reactions involved. AD can be subdivided into extrinsic versus intrinsic subtypes, European/American versus Asian subtypes, and early‐onset (pediatric) versus late‐onset (adult) subtypes. While the extrinsic, European/American, and early‐onset subtypes show type 2 inflammation, the intrinsic, Asian, and late‐onset subtypes have additional type 1 and type 3 inflammation.^13^, ^14^

### 6.3 Pathogenesis of acute eczema: focusing on spongiosis

**FIGURE 7:**
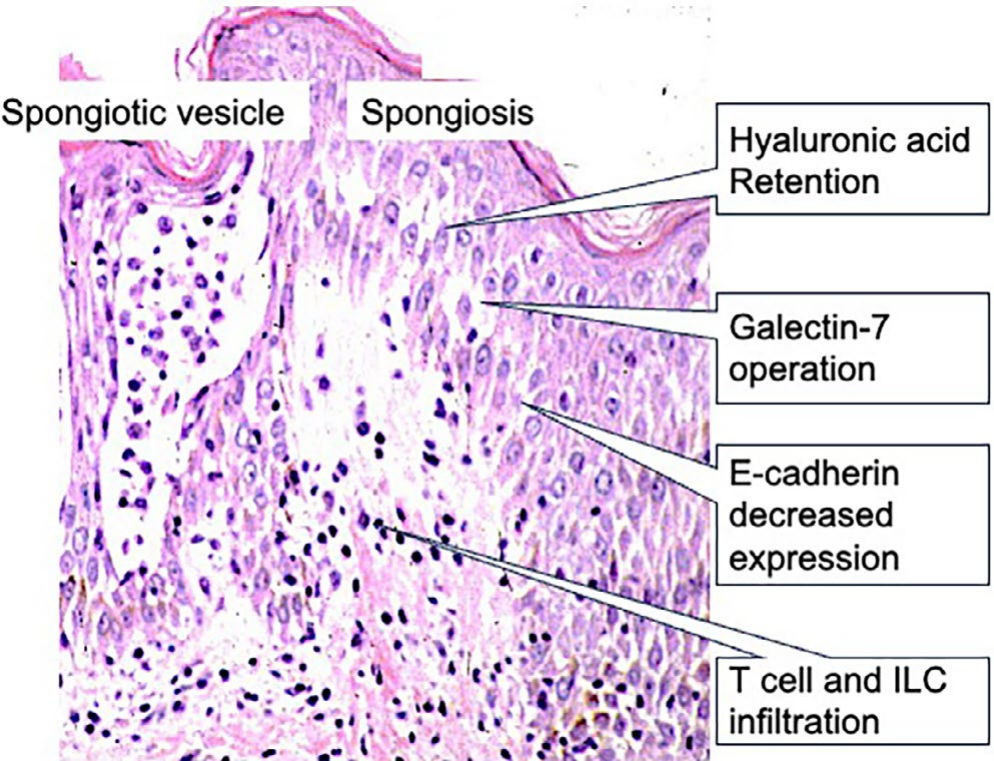
Histopathology and mechanisms of spongiosis in acute eczema. Intercellular edema, called spongiosis, evolves into spongiotic vesicles. In the intercellular spaces, hyaluronic acid is secreted and galectin‐7 is produced by keratinocytes. E‐cadherin expression is decreased to allow fluid to retain. In the upper dermis, various populations of T cells and innate lymphoid cells (ILCs) infiltrate, depending on eczematous diseases. (original magnification x400).

The above findings reveal that acute eczema arises in both immunological states of type 1 and type 2. In consistent with these findings, an interesting study reported that in spongiosis, hyaluronic acid accumulates in the intercellular space of the epidermis, and E‐cadherin expression is decreased^22^ (Figure 7). Given that hyaluronic acid has water‐retaining abilities,^66^ its increased presence contributes to the formation of spongiosis. In this context, the decreased expression of E‐cadherin facilitates fluid retention in keratinocytes. Consistent with the finding that eczema is manifested by both contact dermatitis and AD, IFN‐γ, and IL‐4/IL‐13 increase the production of hyaluronic acid and decrease the expression of E‐cadherin.^22^ Thus, spongiosis can be formed both type 1 and type 2 immune responses.

Galectin‐7 has been shown to be highly expressed in the stratum corneum and intercellular space of the epidermis of AD lesions. A positive correlation has been noted between serum galectin‐7 level and transepidermal water loss in patients with AD.^67^ IL‐4/IL‐13 facilitates the extracellular release of endogenous galectin‐7 in both monolayered human epidermal keratinocytes and three‐dimensional–reconstructed epidermis. This machinery was caused by IL‐4/IL‐13–induced cell damage. Endogenous galectin‐7 serves as a protector from the IL‐4/IL‐13–induced spongiosis model, i.e. disruption of cell‐to‐cell adhesion and/or cell‐to‐extracellular matrix adhesion.^67^ Thus, the feedback that depresses eczema operates in parallel with spongiosis.

### 6.4 Pathogenesis of chronic eczem: focusing on fibrosis and pigmentation

Tissue remodeling takes place along with the healing process of chronic eczema. This process is histologically represented by fibrosis. Periostin has been shown to influence tissue remodeling, fibrosis, regeneration, and repair. Periostin contributes to collagen fibrillogenesis, collagen cross‐linking, and the formation of extracellular matrix.^68^ In allergic reactions, periostin is involved in type 2 immunity and can be induced by IL‐4 and IL‐13.^68^, ^69^ Type 2 cytokine–induced periostin acts on keratinocytes to produce inflammatory cytokines that further enhance the type 2 response, thereby sustaining and amplifying chronic allergic inflammation.^68^ A variety of different allergic diseases, such as bronchial asthma and AD, are associated with periostin expression.^68^, ^69^

In addition to periostin, osteopontin plays multiple roles in the regulation of allergic responses, including regulation of IgE response, inflammatory cell migration, and the development of airway fibrosis and angiogenesis.^70^ Osteopontin has been increasingly recognized for its involvement in the progression of pulmonary, hepatic, and cardiac fibrosis.^71^ Osteopontin is also involved in skin fibrosis associated with systemic sclerosis,^72^ wound healing, and keloid.

IL‐13 is a major inducer of fibrosis in many chronic infectious and autoimmune diseases. IL‐13 induces transforming growth factor β1 in macrophages through a two‐stage process involving, first, the induction of a receptor formerly considered to function only as a decoy receptor, IL‐13Rα2.^73^ It involves IL‐13 signaling through IL‐13Rα2 to activate an AP‐1 variant, which then activates the *TGFB1* promoter.

**FIGURE 8:**
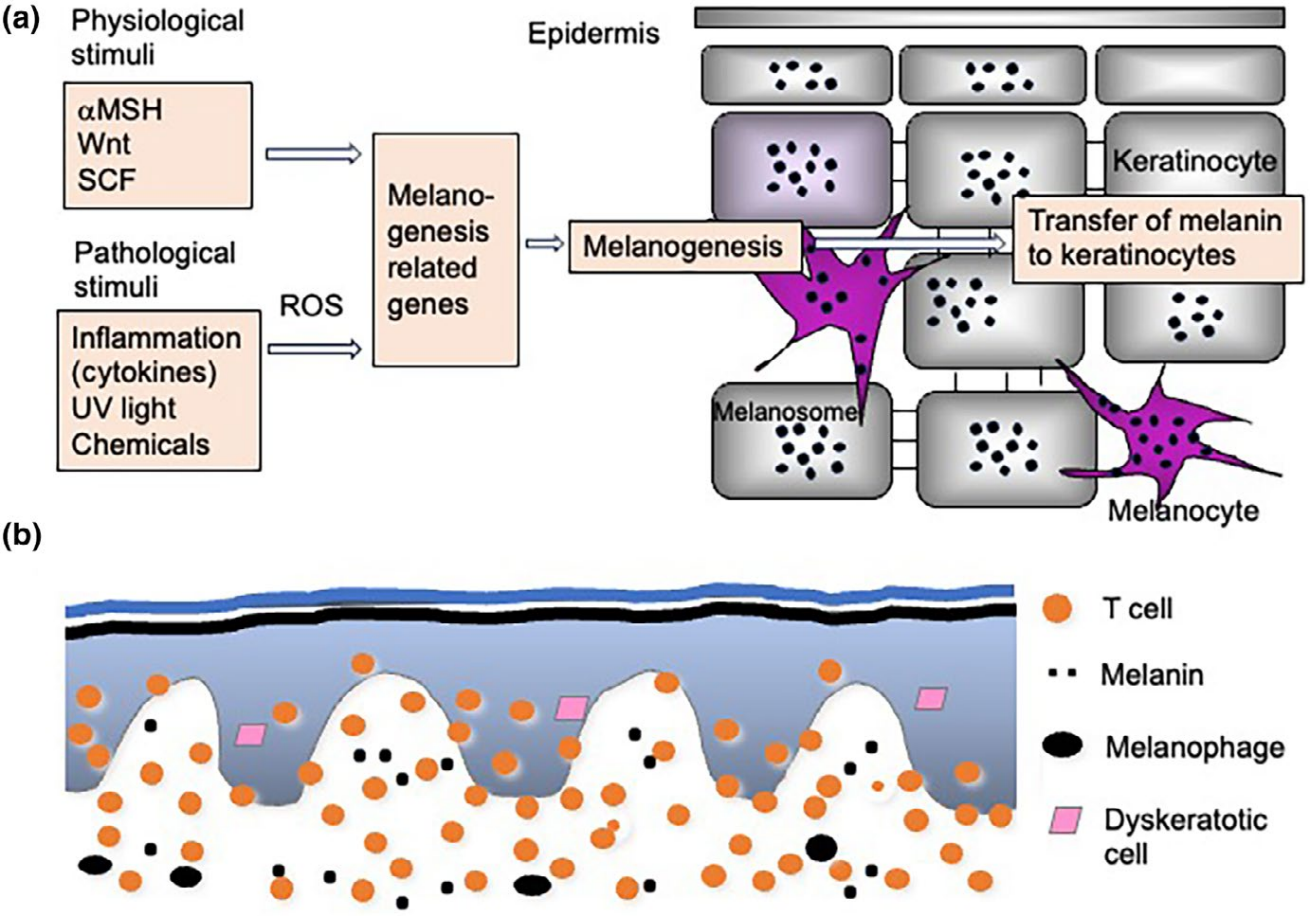
Mechanisms of hyperpigmentation in chronic eczema. Two types of mechanism underlie postinflammatory pigmentation. (a) Instead of a physiological state, pathological stimuli, such as inflammation, induce the expression of melanogenesis‐related genes via reactive oxygen species (ROS). The synthesized melanin pigment is transferred from melanocytes to epidermal keratinocytes. (b) In the inflammatory condition, CD8^+^ T cells attack melanocytes as well as keratinocytes, and melanin is scattered in the upper dermis. Macrophages phagocytose melanin, forming melanophages. α‐MSH, α‐melanocyte–stimulating hormone; SCF, stem cell factor.

Besides fibrosis, hyperpigmentation following inflammation is a common condition in dermatology. In general, there have been two proposed mechanisms underlying postinflammatory hyperpigmentation.^74^ One is increased melanogenesis in the epidermis (Figure 8a) and the other is deposition of melanin pigment in the dermis (Figure 8b).

Physiologically, α‐melanocyte–stimulating hormone, Wnt, and stem cell factor eventually activate microphthalmia‐associated transcription factor, which is the master regulator of melanogenesis, thereby stimulating tyrosinase, tyrosinase‐related protein (TRP) 1, and TRP2^75^, ^76^ (Figure 8a). Tyrosine is changed sequentially to Dopa, Dopaquinone, and eumelanin by tyrosinase.^75^ Melanin pigment is finally transferred to keratinocytes.

Pathologically, inflammation, where cytokines are deeply involved, increases the expression of the melanogenesis‐related genes and eventually leads to melanogenesis.^75^ There is a cascade that consists of inflammation, signal transduction, melanogenesis‐related gene expression, and activation of melanogenesis‐inducing enzymes. A variety of inflammatory factors, such as cytokines, may activate the melanogenesis‐related genes or regulate skin pigmentation processes.^76^ A number of cytokines secreted from T cells, keratinocytes, and fibroblasts may induce melanogenesis, but some are inhibitory. It is interesting that IFN‐γ increases and IL‐4 decreases melanogenesis,^76^ implying that type 1 eczema and type 2 eczema might have opposite effects on hyperpigmentation.

In inflammatory processes, T cells attack melanocytes as well as keratinocytes (Figure 8b). From the damaged melanocytes, melanin pigment is released and scattered in the upper dermis. Macrophages phagocytose the pigment, forming melanophages. This process is typically seen in lichen planus and related diseases called lichenoid eruptions.^77^ Likewise, but to a lesser degree, eczema possibly results in pigment deposition as seen in pigmented contact dermatitis.^78^

### 6.5 Significance of defense systems in eczema

Acute and chronic eczema have different pathophysiological significance for defense towards external invaders. Acute eczema provides measures to eliminate contactants and antigens exposed to the skin. The exudative change with spongiosis may be effective to eliminate stimulants or antigens that penetrate through the stratum corneum barrier.

On the other hand, chronic eczema exhibits a different strategy for defense against external stimuli. The histopathologically observed acanthosis and hyperkeratosis can protect from frequent scratching that destructs the skin barrier. The proliferation of epidermal keratinocytes is induced by IL‐22 and helpfully by IL‐17, which are produced by Th17 and/or Th22 cells. Therefore, type 3 immunity is involved in the development of chronic eczema. IL‐17 and IL‐22 also stimulate keratinocytes to produce human β‐defensin‐2 and cathericidin LL‐37.^79^, ^80^ The epidermal defense is thus reconstructed morphologically by thick epidermis and functionally by increased antimicrobial peptides. In eczema, the above events operate sequentially and gradually so that the boundary between acute and chronic eczema is continuous.

Similarly to chronic eczema, type 3 inflammation is also observed in AD^64^, ^65^, ^81^ and prurigo nodularis.^82^ In AD subtypes, extrinsic versus intrinsic, European/American versus Asian, and early‐onset versus late‐onset AD,^13^, ^14^ while extrinsic, European/American and early‐onset AD show relatively pure type 2 inflammation, intrinsic, Asian, and late‐onset AD have additional type 3 and/or type 1 inflammation.^14^ These types seem to be led by virtually the same immunological mechanism as chronic eczema.

### 6.6 Eczema and innate immunity

Chemical sensitizers activate innate immune cells, which orchestrate the skin immune response. This involves oxidative and inflammatory pathways as an initial step. In parallel, the Nrf2/Keap1 pathway, a suppressor for cellular oxidative and electrophilic stress, is activated in the different skin innate immune cells including epidermal Langerhans cells and dermal dendritic cells, but also in keratinocytes.^83^ Subsequently, activation of the antioxidant response element protects from oxidative stress. In addition to the chemical protein‐binding assay, this antioxidant response element assay is also used for the prediction of the sensitizing ability of chemicals in keratinocytes.

The involvement of ILCs in eczema has more recently been suggested. In type 1 dominant allergic contact dermatitis, the balance between ILC1 and ILC2 is skewing to ILC1.^46^ The critical role of ILC2 has been extensively studied in AD.^50^, ^51^ ILC2, producing IL‐13, IL‐5, IL‐4, and IL‐31, is directly stimulated by alarmins, IL‐33, IL‐25, and TSLP, derived from epidermal keratinocytes.^51^ The alarmins also stimulate basophils to produce IL‐4, thereby further activating ILC2.^51^ The activation of ILC2 is currently considered crucial in the pathogenesis of AD. While the skin lesions of AD are clinically exaggerated without specific antigens, some stimuli towards the epidermis can flare up eczema in an antigen‐nonspecific manner. This suggests that Th2 cells are not necessarily required for AD pathology, but certain immune cells without a T‐cell receptor, such as ILC2, exert a waxing action.

Thus, it seems that innate immunity, in particular ILC2, is highly involved in AD, compared with contact dermatitis. Since both are representative eczematous diseases, the extent of involvement of innate immunity depends on individual diseases.

## 7 FUTURE PERSPECTIVES

Approximately 120 years have passed since the appearance of the eczema triangle in Kreibich's textbook. Eczema remains not fully elucidated, and, even currently, it provides one of the attractive themes in dermatology. Clinically, eczema manifests as various phenotypic aspects, which are altered chronologically in each patient. Many populations of T cells and ILCs are involved in the pathogenesis, depending on individual eczematous diseases.

While exquisite mechanisms are present in the skin lesion of eczema, the inflammation may be accompanied by systemic disorders. It has recently been reported that inflammatory skin diseases are associated with an increased risk of comorbidities. Psoriasis is a notable representative disease, and comorbidities include arthritis, Crohn disease, obesity, and cardiovascular diseases. These factors have a significant impact on the decision to use one therapy over another.^84^ Historically, patients with severe eczema tend to experience food allergies, but this association has currently been described as a causative role of skin barrier impairment in relation to AD.^85^ On the other hand, some studies have suggested a positive association between eczema and cardiovascular disease, probably through enhanced systemic inflammation. Self‐reported frequent eczema was associated with increased risk of mortality from coronary heart disease, but not other major cardiovascular disease, in a Japanese general population.^86^

In another line of investigation on eczema comorbidities, several studies were conducted to investigate the association between eczema and risk of depression. In an investigation using 10 studies with a total of 188 495 patients, the random‐effects model summarizing all comparisons suggested a positive association between eczema and risk of depression.^87^ Similar results were observed in subgroup analysis by region. This investigation showed that patients with eczema were associated with an increased risk of depression. Patients with the gain‐of‐function mutation of Janus kinase 1 exhibit AD or severe eczema, bronchial asthma, hypereosinophilia, food and environmental allergies,^88^, ^89^, ^90^ and autism.^90^ The JAK–STAT signaling pathway is associated with many neurodevelopmental disorders,^91^ as well as allergic disorders, suggesting the relationship between eczema and mental conditions. It is unknown whether eczema is associated with systemic diseases due to its inflammatory characteristics.

Eczema has recently been investigated by means of modern science technologies. The single‐cell RNA sequencing method allows us to examine peripheral blood mononuclear cells^92^ and skin‐infiltrating immune cells precisely.^93^ Alterations in innate immune cells and impaired cytotoxic cells can be observed in severe AD.^93^ The impact of these alterations requires further investigation. Future studies will further clarify the disease pathogenesis and effectiveness of potential therapeutic targets in eczematous diseases.

## FUNDING INFORMATION

None.

## CONFLICT OF INTEREST STATEMENT

The authors declare no conflicts of interest.
